# Prediction of red blood cell transfusion after orthopedic surgery using an interpretable machine learning framework

**DOI:** 10.3389/fsurg.2023.1047558

**Published:** 2023-03-02

**Authors:** Yifeng Chen, Xiaoyu Cai, Zicheng Cao, Jie Lin, Wenyu Huang, Yuan Zhuang, Lehan Xiao, Xiaozhen Guan, Ying Wang, Xingqiu Xia, Feng Jiao, Xiangjun Du, Guozhi Jiang, Deqing Wang

**Affiliations:** ^1^ School of Public Health (Shenzhen), Shenzhen Campus of Sun Yat-sen University, Shenzhen, China; ^2^ Department of Transfusion Medicine, The First Medical Center of Chinese PLA General Hospital, Beijing, China; ^3^ The Second School of Clinical Medicine, Guangdong Medical University, Dongguan, China; ^4^ HealSci Technology Co., Ltd, Beijing, China; ^5^ Guangzhou Centre for Applied Mathematics, Guangzhou University, Guangzhou, China; ^6^ School of Public Health (Shenzhen), Sun Yat-sen University, Guangzhou, China

**Keywords:** orthopedic surgery, RBC transfusion, prediction model, machine learning, interpretability

## Abstract

**Objective:**

Postoperative red blood cell (RBC) transfusion is widely used during the perioperative period but is often associated with a high risk of infection and complications. However, prediction models for RBC transfusion in patients with orthopedic surgery have not yet been developed. We aimed to identify predictors and constructed prediction models for RBC transfusion after orthopedic surgery using interpretable machine learning algorithms.

**Methods:**

This retrospective cohort study reviewed a total of 59,605 patients undergoing orthopedic surgery from June 2013 to January 2019 across 7 tertiary hospitals in China. Patients were randomly split into training (80%) and test subsets (20%). The feature selection method of recursive feature elimination (RFE) was used to identify an optimal feature subset from thirty preoperative variables, and six machine learning algorithms were applied to develop prediction models. The Shapley Additive exPlanations (SHAP) value was employed to evaluate the contribution of each predictor towards the prediction of postoperative RBC transfusion. For simplicity of the clinical utility, a risk score system was further established using the top risk factors identified by machine learning models.

**Results:**

Of the 59,605 patients with orthopedic surgery, 19,921 (33.40%) underwent postoperative RBC transfusion. The CatBoost model exhibited an AUC of 0.831 (95% CI: 0.824–0.836) on the test subset, which significantly outperformed five other prediction models. The risk of RBC transfusion was associated with old age (>60 years) and low RBC count (<4.0 × 10^12^/L) with clear threshold effects. Extremes of BMI, low albumin, prolonged activated partial thromboplastin time, repair and plastic operations on joint structures were additional top predictors for RBC transfusion. The risk score system derived from six risk factors performed well with an AUC of 0.801 (95% CI: 0.794–0.807) on the test subset.

**Conclusion:**

By applying an interpretable machine learning framework in a large-scale multicenter retrospective cohort, we identified novel modifiable risk factors and developed prediction models with good performance for postoperative RBC transfusion in patients undergoing orthopedic surgery. Our findings may allow more precise identification of high-risk patients for optimal control of risk factors and achieve personalized RBC transfusion for orthopedic patients.

## 1. Introduction

Orthopedic surgery is usually related to complicated operations and deep cuts, which results in orthopedic patients having a high risk of intraoperative bleeding and difficulty in stopping the bleeding (1, 2), whilst allogeneic blood transfusion (ABT) is often administered to patients undergoing orthopedic surgery (3, 4). Red blood cell (RBC) is most commonly used when surgical patients need ABT. Although allogeneic RBC transfusion can improve the health of patients (5), it might be accompanied by many side effects, such as surgical site infections and multiple complications, thus adversely leading to physical deterioration (6, 7). Previous studies have also shown that receiving an allogeneic blood transfusion is associated with a decrease in survival rate in the short or long term (8).

Identification of risk factors and accurate predictions of RBC transfusion before patients undergo surgical treatment is of great importance in clinical practice. Typically, physicians make decisions of blood transfusion primarily based on their clinical experience and the hemoglobin (Hb) level of the patient (9). However, this strategy often ignores other potential preoperative indicators and may lead to one-sided and inappropriate transfusion. Recent studies have identified other risk factors including BMI, age, blood pressure, or use of medications, and showed that Hb might not be the most critical risk factor in postoperative RBC transfusion (10, 11). Therefore, more modifiable factors should be considered before the RBC transfusion decision is made. Furthermore, most studies on the prediction of postoperative RBC transfusion have been based on traditional statistical models, which are often required to meet a series of strict assumptions, such as additive and linearity. However, these assumptions may not always hold in the real world. In addition, traditional regression analyses examine the associations of predictors one-by-one or a specific subset based on prior knowledge and neglect the interactive or modifying effects among predictors, which might lead to the omission of significant predictors and loss of prediction performance (12).

Machine learning methods, which use data-driven mathematical models to perform predictions, are widely employed to deal with complicated medical problems, and often exhibit better prediction performance compared with traditional statistical methods, especially in handling enormous numbers of predictors (13, 14). Traditional machine learning methods focus on the accuracy of prediction and are black-box in nature, thus it is difficult to be interpreted biologically. Therefore, an interpretable machine learning framework was developed to explain the output of prediction models (15). If the outcome of RBC transfusion is accurately predicted before surgery, interventions can be taken to prevent complications for patients at high risk of postoperative transfusion, whereas inappropriate transfusion of RBC would increase the risk of surgical errors and deficient blood supply, or lead to waste of blood resources (16). Different machine learning models have been applied to the prediction of blood transfusion, such as XGBoost and tree-based models (17, 18). However, to the best of our knowledge, there is no study to predict postoperative RBC transfusion in patients with orthopedic surgery using machine learning approaches.

In this study, we aimed to identify the risk factors of postoperative RBC transfusion in orthopedic patients and further establish prediction models of RBC transfusion after orthopedic surgery based on an interpretable machine learning framework.

## 2. Materials and methods

### 2.1. Study population

**Figure 1:**
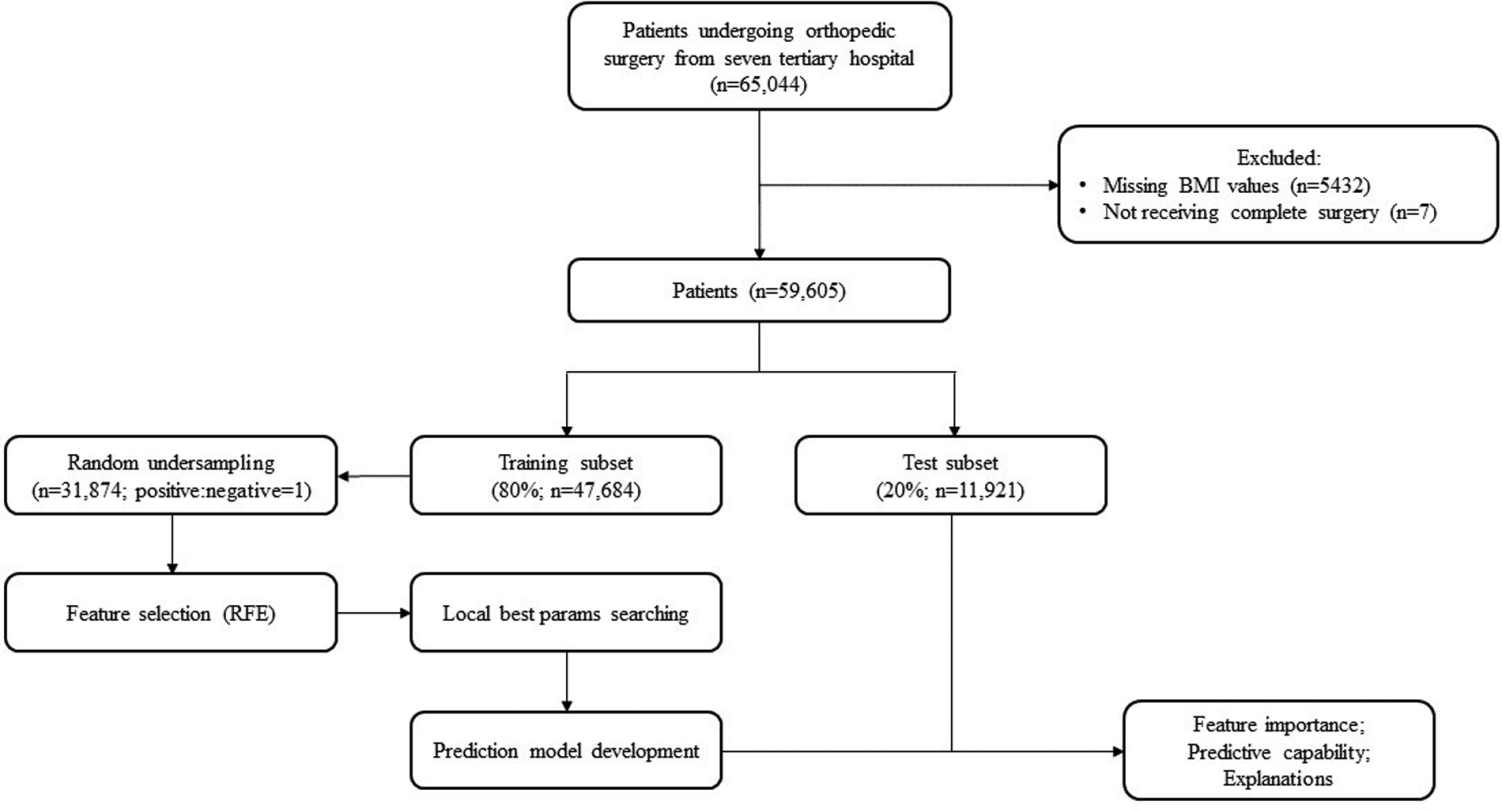
Flowchart of the study design.

Data were retrieved from the National Preoperative Anemia Database, which was initiated by The First Medical Center of People's Liberation Army (PLA) General Hospital. From June 2013 to January 2019, electronic medical records of 65,044 patients with orthopedic surgery were collected at 7 tertiary hospitals located in China. Variables were gathered retrospectively, including demographics of patients, and clinical and hematology information. The recruitment methods, deﬁnitions, and biochemical investigations have been described in detail elsewhere (19). After excluding 5,432 patients with missing values of BMI and 7 patients who did not complete surgery, 59,605 individuals were included in our study (Figure 1). This study was approved by the Medical Ethics Committee of the Chinese PLA General Hospital (approval number, S2018–245–01), and followed RECORD guidelines for data collection.

### 2.2. Clinical and laboratory measurements

All variables were collected from the medical record systems of the seven hospitals. A total of thirty preoperative variables were incorporated into our study. The information of sex, age, and other demographics was recorded at hospital admission. BMI was calculated from the weight and height of patients. Hematology parameters including RBC count, Hb, and albumin (ALB) level were also measured before orthopedic surgery. For preoperative laboratory indicators which were measured more than once, we used the values closest to the start time of surgery. To make laboratory indicators comparable among different hospitals, all the data were required to pass internal quality control and external quality control evaluation organized by the National Health Commission of the People's Republic of China (19). The surgery procedures were classified into ten categories by the criterion of ICD-9-CM-3, according to the top two codes.

The designated physician of each member hospital extracted the qualified data of inpatient surgical procedures from the Hospital Information System, and then uploaded the data to the platform annually after de-identification. Data quality is ensured through comprehensive training of the physicians, an audit of the participating hospital, regular conference calls, and an annual training meeting. Contribution to the National Preoperative Anemia Database is voluntary.

### 2.3. Definition of clinical outcomes

According to the transfusion information of the status and time of RBC transfusion and the surgical information, the primary outcome in this study was defined as the reception of postoperative RBC transfusion. The RBC transfusion group was defined as patients receiving at least 1 unit RBC transfusion from postoperation to discharge.

### 2.4. Machine learning and statistical analysis

The data were randomly split into a training subset and a test subset, and the ratios of cases and controls were maintained across the two subsets. 80% data of patients were used to develop prediction models, whilst the other 20% of patients were used as validation. Recursive feature elimination (RFE), a feature selection method, was used to identify an optimal subset of predictors. RFE algorithm cyclically eliminates features with the lowest importance in the training set until they reached a specified termination criterion, such as the maximized accuracy or a preset AUC threshold (20). The subset which had the largest AUC would be assigned to the built prediction model. The resampling method of random undersampling was employed to balance the disproportion of outcome distribution. Six commonly used machine learning algorithms, including Logistic Regression (LR), K-Nearest Neighbors (KNN), Support Vector Machine (SVM), Random Forest (RF), eXtreme Gradient Boosting (XGBoost), and Gradient Boosting with Categorical Features Support (CatBoost) were employed to develop prediction models. The prediction performance was quantified by calculating the accuracy, precision, recall, f1-score, AUC, and Matthews correlation coefficient (MCC) of models.

An interpretable machine learning framework was used to explain the outputs of the prediction models. The SHapley Additive exPlanations (SHAP) method was used to provide consistent and locally accurate attribution values for each predictor in the prediction model (21). A higher SHAP value represents a larger contribution of a predictor toward the prediction. SHAP approach provided a partial dependence plot to graphically present the linear or nonlinear relationship between each predictor and the outcome.

Although the interpretable approach was applied to explain the output of the machine learning model, a relatively large number of predictors and the complexity of the model limited its application. For simplicity of the clinical utility, therefore, a risk score system was further established. To quantify the risk effect of predictors, a multivariable logistic regression model was created. Those variables, which were selected by the RFE algorithm but had a relatively minor contribution to the prediction model (accounting for <5% of total feature importance), were excluded to simplify the risk score system. Moreover, continuous variables were discretized before model establishment. The discretization thresholds were determined by clinical reference ranges or the results of partial dependence plots. Each variable was assigned a score according to the effect size derived from the logistic model, and the sum of scores reflected the magnitude of risk of postoperative RBC transfusion. The prediction performance of the risk score model was also evaluated on both training and test sets.

All data were expressed as percentages, means, and SDs, or medians and interquartile ranges (IQRs) as appropriate. Pearson chi-squared test or Fisher's exact test for categorical variables, and Student t-test or Mann-Whitney *U* test for continuous variables were used as appropriate. All statistical tests were 2-sided, and a *P*-value < 0.05 was considered to be significant. All the analysis was completed using Python 3.8.

## 3. Results

### 3.1. Baseline characteristics of the study cohort

The study cohort consisted of 59,605 patients (mean age 50.50 ± 18.08 years, 51.45% males, mean BMI 24.11 ± 3.74 kg/m^2^), 47,684 (80%) allocated to the training subset, and 11,921 (20%) to the test subset (Figure 1). Of the 59,605 patients, 19,921 (33.40%) underwent RBC transfusion after orthopedic surgery. The most common operation was arthrosis repair and plastics (33.16%). Compared with patients without RBC transfusion, those with transfusion were more likely to be older, had a lower RBC count, Hb level, ALB level, and longer activated partial thromboplastin time (APTT) (Table 1).

**Table 1:** Main preoperative characteristics of the study population of two groups (receiving postoperative RBC transfusion or not).

Variates	No RBC Transfusion Group	RBC Transfusion Group	*P-*value
Age (yrs), mean ± SD	47.28 ± 17.36	56.91 ± 17.76	<0.001
BMI (kg/m^2^), mean ± SD	23.87 ± 3.50	24.60 ± 4.13	<0.001
Albumin (g/L), mean ± SD	42.05 ± 3.93	39.82 ± 4.30	<0.001
Alanine Aminotransferase (U/L), median (IQR)	17.60 (12.20–27.37)	15.80 (11.30–24.20)	<0.001
Activated Partial Thromboplastin Time(sec), mean ± SD	33.84 ± 5.77	36.01 ± 6.07	<0.001
Aspartate Aminotransferase (U/L), median (IQR)	17.10 (14.10–21.80)	16.70 (13.80–21.30)	<0.001
Creatinine(*μ*mol/L), median (IQR)	67.40 (57.00–79.40)	63.40 (54.20–74.70)	<0.001
Hemoglobin(g/L), mean ± SD	136.96 ± 18.67	126.71 ± 18.70	<0.001
Hematocrit (%), mean ± SD	40.45 ± 5.07	37.78 ± 5.18	<0.001
Mean Corpuscular Hemoglobin (pg), mean ± SD	30.25 ± 2.07	29.97 ± 2.28	<0.001
Mean Corpuscular Hemoglobin Concentration (g/L), mean ± SD	338.18 ± 12.29	335.19 ± 12.36	<0.001
Mean Corpusular Volume (fl), mean ± SD	89.45 ± 5.24	89.37 ± 5.67	0.373
Platelet (10^9^/L), median (IQR)	224.00 (187.00–267.00)	227.00 (186.00–274.25)	<0.001
Prothrombin Time(sec), mean ± SD	12.70 ± 1.72	13.13 ± 1.31	<0.001
Red Blood Cell Count (10^12^/L), mean ± SD	4.59 ± 0.53	4.25 ± 0.60	<0.001
Total Bilirubin (μmol/L), median (IQR)	10.70 (8.00–14.30)	10.30 (7.80–13.70)	<0.001
White Blood Cell Count(10^9^/L), median (IQR)	6.63 (5.47–8.22)	6.26 (5.18–7.77)	<0.001
Sex, *N* (%)			<0.001
Male	22,560 (37.85%)	8,108 (13.60%)	
Female	17,124 (28.73%)	11,813 (19.82%)	
Iron Supplements Use, *N* (%)			<0.001
No	38,581 (64.73%)	17,787 (29.84%)	
Yes	1,103 (1.85%)	2,134 (3.58%)	
Erythropoietin Use, *N* (%)			<0.001
No	31,641 (53.08%)	14,724 (24.70%)	
Yes	1,609 (2.70%)	3,472 (5.83%)	
Folic Acid Use, *N* (%)			<0.001
No	33,229 (55.75%)	18,160 (30.47%)	
Yes	21 (0.04%)	36 (0.06%)	
Vitamin B12 Use, *N* (%)			<0.001
No	33,240 (55.77%)	18,193 (30.52%)	
Yes	10 (0.02%)	3 (0.01%)	
In-hospital Times, *N* (%)			<0.001
1	31,410 (52.70%)	15,144 (25.41%)	
>1	4,845 (8.13%)	3,901 (6.54%)	
Operation Type, *N* (%)			<0.001
Incision, excision, and division of other bones	2,684 (4.50%)	1,483 (2.49%)	
Other operations on bones, except facial bones	2,616 (4.39%)	304 (0.51%)	
Reduction of fracture and dislocation	9,092 (15.25%)	2,756 (4.62%)	
Incision and excision of joint structures	7,590 (12.73%)	1,921 (3.22%)	
Repair and plastic operations on joint structures	9,101 (15.27%)	10,663 (17.89%)	
Operations on muscle, tendon, and fascia of hand	423 (0.71%)	1 (0.00%)	
Operations on muscle, tendon, fascia, and bursa, except hand	2,231 (3.74%)	308 (0.52%)	
Other procedures on musculoskeletal system	260 (0.44%)	218 (0.37%)	
Operations and procedures on other systems	5,687 (9.54%)	2,267 (3.80%)	

SD = standard deviation; IQR = Interquartile range.

### 3.2. Interpretable machine learning model

**Figure 2:**
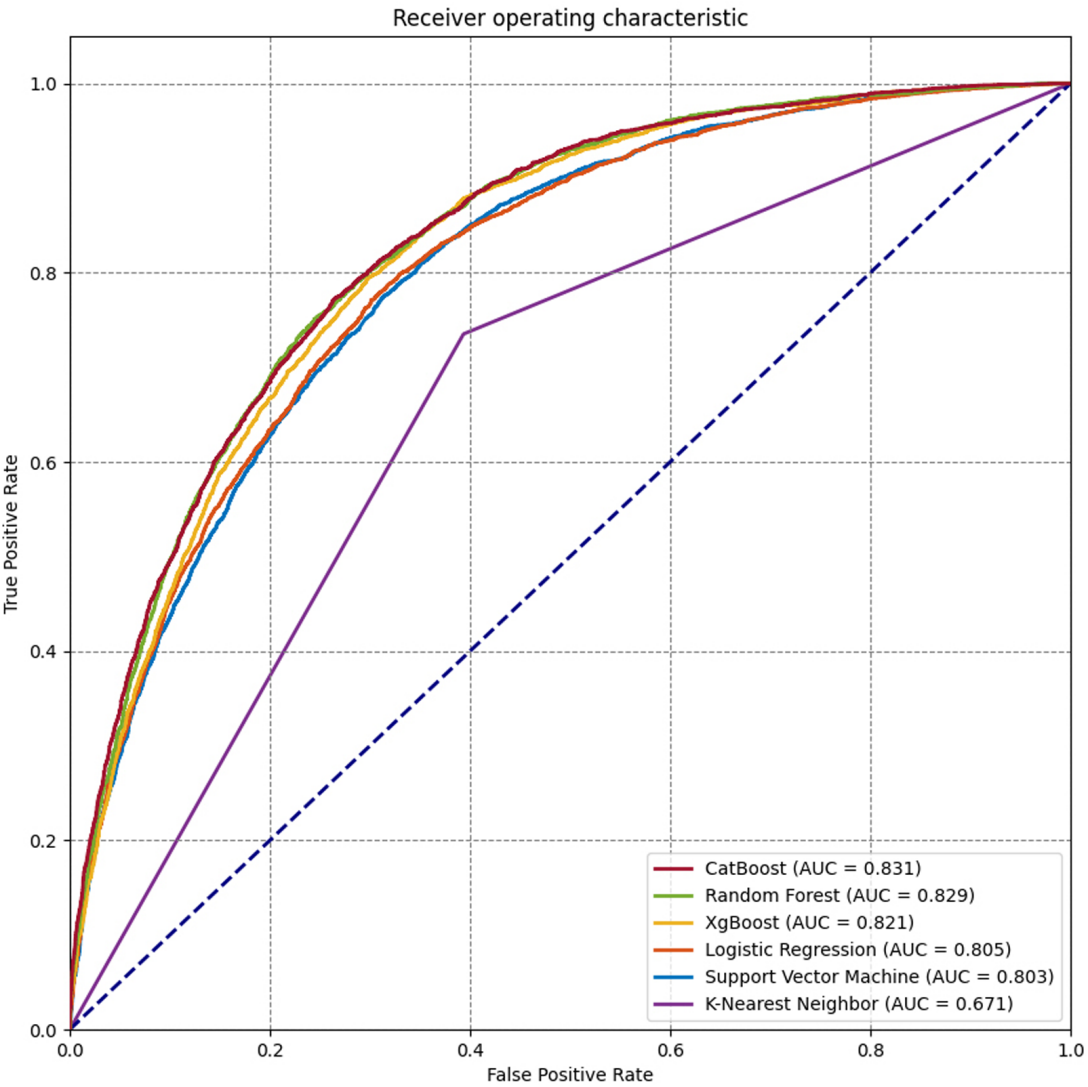
Receiver operating characteristic (ROC) curves for the machine learning and logistic regression prediction models.

Seventeen variables were identified as important predictors by the RFE algorithm based on the criterion of AUC (Supplementary Figure S1), including age, sex, BMI, type of operation, ALB, alanine aminotransferase (ALT), APTT, aspartate aminotransferase (AST), creatinine (CR), Hb, hematocrit (HCT), mean corpuscular hemoglobin concentration (MCHC), platelet (PLT), prothrombin time (PT), RBC count, white blood cell (WBC) count, and use of erythropoietin. A series of prediction models were established using different machine learning algorithms. As shown in Figure 2, the model derived from the CatBoost algorithm had the best prediction performance compared with the other five models, with an AUC of 0.864 (95% CI: 0.861–0.867) and 0.831 (95% CI: 0.824–0.836) on the training and test subsets, respectively (Table 2). The accuracy and precision of the CatBoost model on the test subset were 0.766 and 0.656, respectively, which were higher than other prediction models.

**Table 2:** The prediction performance of different machine learning models in the training subset and test subset.

	Model	Accuracy	Precision	Recall	F1 score	AUC (95% CI)	MCC
Training Subset	CatBoost	0.763	0.824	0.670	0.739	0.864 (0.861–0.867)	0.536
	XGBoost	0.766	0.752	0.792	0.771	0.840 (0.837–0.844)	0.532
	RF	0.821	0.807	0.844	0.825	0.821 (0.817–0.824)	0.643
	LR	0.731	0.733	0.728	0.730	0.731 (0.727–0.735)	0.462
	SVM	0.728	0.731	0.721	0.726	0.728 (0.724–0.732)	0.456
	KNN	0.669	0.650	0.735	0.690	0.669 (0.665–0.674)	0.342
Test Subset	CatBoost	0.766	0.656	0.633	0.644	0.831 (0.824–0.836)	0.471
	XGBoost	0.737	0.580	0.774	0.663	0.821 (0.814–0.827)	0.467
	RF	0.741	0.584	0.782	0.669	0.830 (0.823–0.835)	0.477
	LR	0.721	0.560	0.773	0.649	0.805 (0.798–0.812)	0.443
	SVM	0.721	0.563	0.742	0.640	0.803 (0.796–0.809)	0.430
	KNN	0.650	0.484	0.735	0.584	0.671 (0.663–0.678)	0.322

RF, random forest; LR, logistic regression; SVM, support vector machine; KNN, k-nearest neighbor; AUC, the area under the operating characteristic curve; CI, confidence interval; MCC, Matthews correlation coefficient.

**Figure 3:**
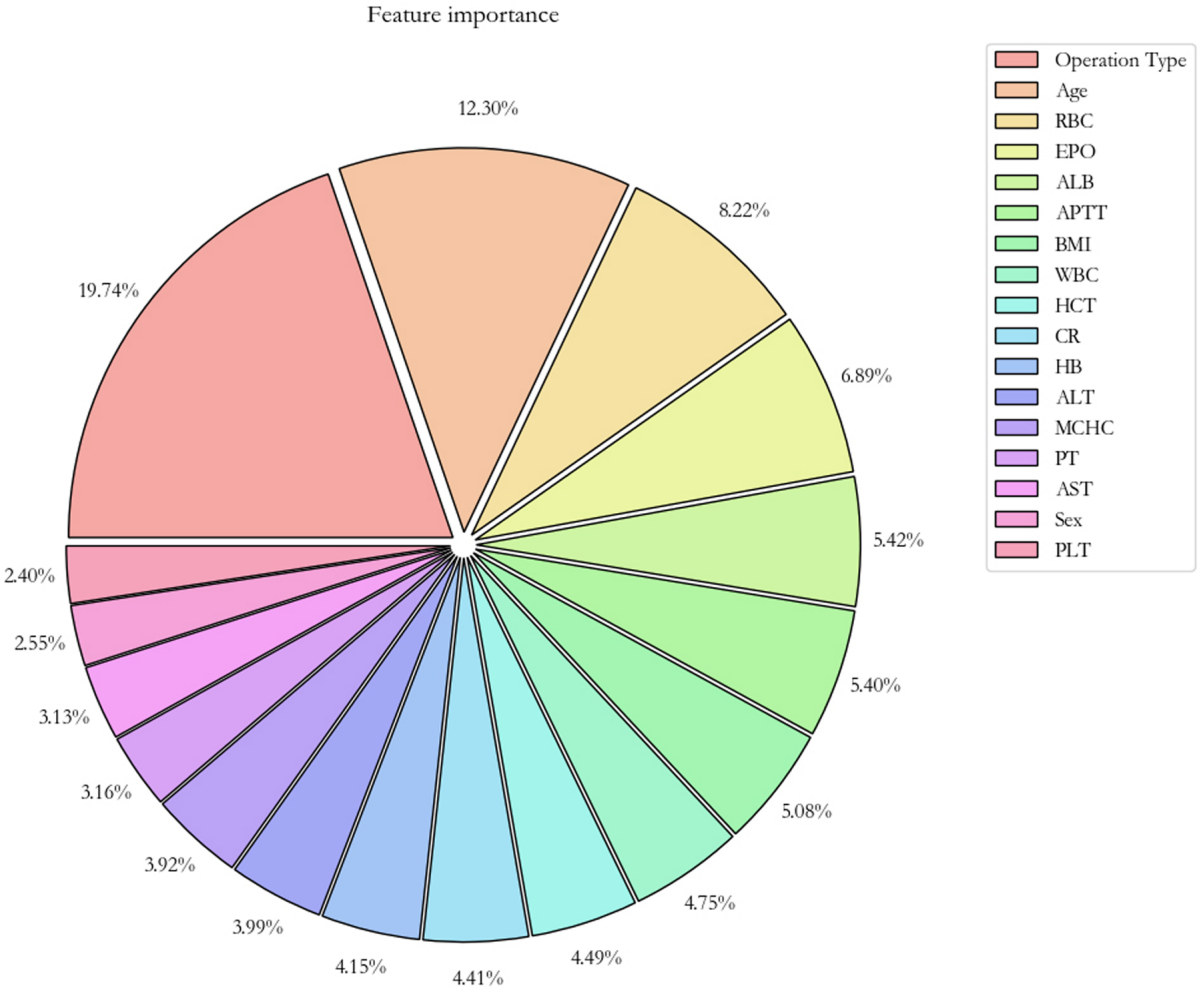
Importance of the features in the catBoost prediction model. The pie plot shows the relative importance of features for predicting the risk of postoperative RBC transfusion, sorted by importance from high to low. RBC, red blood cell count; EPO, erythropoietin use; ALB, albumin; APTT, activated partial thromboplastin time; WBC, white blood cell count; HCT, hematocrit; CR, creatinine; Hb, hemoglobin; ALT, alanine aminotransferase; MCHC, mean corpuscular hemoglobin concentration; PT, prothrombin time; AST, aspartate aminotransferase; PLT, platelet.

**Figure 4:**
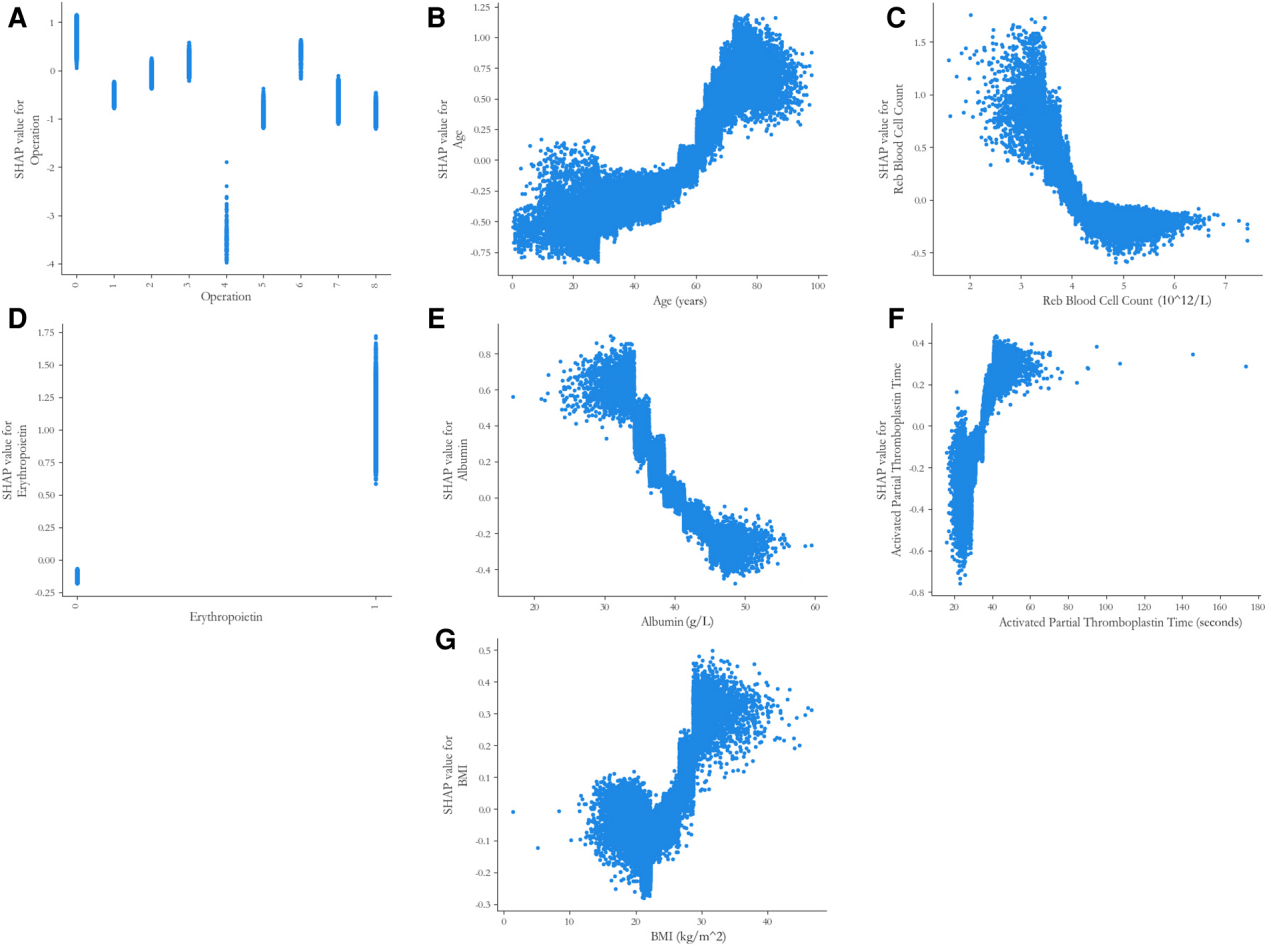
SHAP partial dependence plots of the five predictors in the catBoost model. Each plot shows how a single risk factor affects the outcome of the prediction model. SHAP values for specific factors exceed zero, representing an increased risk of postoperative RBC transfusion in orthopedic surgery patients. SHAP, SHapley Additive exPlanations. (**A**) Code on the x-axis: 0 = Repair and plastic operations on joint structures; 1 = Incision and excision of joint structures; 2 = Operations and procedures on other systems; 3 = Incision, excision, and division of other bones; 4 = Operations on muscle, tendon, and fascia of hand; 5 = Operations on muscle, tendon, fascia, and Bursa, except hand; 6 = Other procedures on musculoskeletal system; 7 = Reduction of fracture and dislocation 8 = Other operations on bones, except facial bones; (**D**) Code on the x-axis: 0 = no use, 1 = use.

With respect to the feature importance of the CatBoost model, there were seven predictors, including the type of operation, age, RBC count, preoperative erythropoietin, ALB level, APTT, and BMI, accounted for >5% of total feature importance (Figure 3). The impact of different indicators on the risk of postoperative RBC transfusion was evaluated by the SHAP approach. As shown in Figure 4A, the risk of postoperative RBC transfusion varied in different orthopedic operations. Patients who underwent repair and plastic operations on joint structures had the highest risk of postoperative RBC transfusion. Among the operations in the orthopedic department, the operations on muscle, tendon, and fascia of hand were associated with the lowest risk of postoperative RBC transfusion. There were non-linear relationships between the risk of postoperative RBC transfusion and age, RBC count, or BMI (Figure 4B,C, G), whereas the relationships for levels of ALB and Hb were approximately linear with a negative slope (Figure 4E, Supplementary Figure S2). Patients with age under 60 years old, RBC count over 4.0 × 10^12^/L, higher ALB level, and BMI between about 18 and 25 had a lower risk of postoperative RBC transfusion. Also, other variables in the model may partly contribute to the outcomes.

### 3.3. Risk score system

For simplicity of clinical application, we further constructed a risk score system using the coefficients of predictors in the logistic regression model. The top six risk factors, including the operation type, age, RBC count, ALB, APTT, and BMI, were incorporated into the risk score model (Table 3), The total score ranged from 0 to 56 theoretically, and patients with a score greater than 28 (mean value of the risk score) would be considered as high-risk individuals. Even with fewer variables compared with the machine learning model, the risk score model still performed relatively well, whose AUCs on the training subset and test subset were 0.776 (95% CI: 0.772–0.781) and 0.778 (95% CI: 0.771–0.785), respectively (Table 4).

**Table 3:** The risk score model based on the *β*-coefficient of logistic regression analysis.

Variables	Beta	Score
Albumin ≤ 40 g/L	0.516	5
Activated Partial Thromboplastin Time > 36 seconds	0.442	4
Red Blood Cell Count ≤ 4.0*10^12^/L	1.035	10
Age > 60 years	0.747	7
BMI (kg/m^2^)		
BMI > 23.0	0.247	2
18.5 < BMI ≤ 23.0	–	0
BMI ≤ 18.5	0.433	4
Operation		
Repair and plastic operations on joint structures	2.625	26
Incision and excision of joint structures	1.142	11
Operations and procedures on other systems	1.653	17
Operations on muscle, tendon, fascia, and bursa, except hand	2.050	21
Other procedures on musculoskeletal system	0.676	7
Operations on facial bones and joints	2.271	23
Reduction of fracture and dislocation	1.187	12
Other operations on bones, except facial bones	0.792	8
Operations on muscle, tendon, and fascia of hand	–	0

**Table 4:** The prediction performance of the risk score model in the training subset and test subset.

	Accuracy	Precision	Recall	F1 score	AUC (95% CI)	MCC
Training Subset	0.709	0.725	0.674	0.698	0.776 (0.772–0.781)	0.419
Test Subset	0.719	0.567	0.679	0.618	0.778 (0.771–0.785)	0.403

AUC, the area under the operating characteristic curve; CI, confidence interval; MCC, Matthews correlation coefficient.

## 4. Discussion

In this multicenter retrospective cohort study, we developed prediction models for the postoperative RBC transfusion of orthopedic patients in the Chinese population based on the interpretable machine learning framework. Our main findings were 3-fold. First, we established a prediction model derived from the CatBoost algorithm, which presented better prediction performance when compared with the other five machine learning models. Second, besides the low level of Hb, we identified old age of orthopedic surgery, low ALB and RBC count, extremes of BMI, prolonged APTT, repair and plastic operations on joint structures, and use of erythropoietin as significant predictors for postoperative RBC transfusion in orthopedic patients. Third, we developed a simple risk score system with fewer predictors but similar prediction performance as machine learning models, which was intuitive and could be used more easily in clinical settings. These findings would enable the identification of high-risk patients for optimal control of risk factors and achieve personalized RBC transfusion for orthopedic patients.

In this analysis, we developed a prediction model for RBC transfusion in orthopedic patients using the machine learning algorithm of CatBoost, which showed a higher AUC of 0.831 when compared with the other five models (LR, KNN, SVM, RF, XGBoost). Compared with the previous prediction model which included perioperative variables, our prediction model only used preoperative indicator, so preventive measures could be taken when clinicians planned operations for patients (22). Moreover, most previous studies on postoperative blood transfusion in orthopedic patients only focusing on a specific type of surgery, rather than incorporating all types of surgery in a clinical department, which limits the application of the prediction model. In addition, some other studies only identify the risk factors of postoperative transfusion but without individual prediction (23, 24). In our prediction model, different types of orthopedic surgery were incorporated, which could be applied in the whole department to help clinicians identify high-risk patients, and further improve the management of postoperative RBC transfusion for patients. Hence, the potentially unnecessary repeat preoperative testing, such as the testing of hematological and biochemical indexes, could be avoided. With consideration of the large number of patients undergoing orthopedic surgery, the saving in medical tests and transfusions would be of benefit to the individual, family, and society to reduce the medical burdens.

The old age of orthopedic surgery, low ALB and RBC count, extremes of BMI, high APTT, operation of repair and plastic operations on joint structures, and use of erythropoietin were identified as important risk factors in our study. The relative feature importance suggested that there were several predictors that contributed more to the prediction of the postoperative RBC transfusion in orthopedic patients compared with that of Hb. Similar findings were reported in previous studies. Raman et al. (10) developed a decision tree-based machine learning model to predict the transfusion units after adult spinal deformity surgery and found that the number of levels fused was the most significant risk factor, whilst the preoperative Hb was not included in the model. In another study on total shoulder arthroplasty, Gowd et al. (25) identified preoperative hematocrit, BMI, and operative time as the most important risk factors of postoperative transfusion and other short-term complications. These results suggest that it may be not appropriate to simply speculate the requirement of postoperative transfusion only based on the levels of Hb, but more risk factors need to be considered, such as age, BMI, and RBC count (26, 27). Even if the preoperative Hb level of the patient is high, other risk factors might significantly contribute to the postoperative transfusion (18). Interestingly, we identified low ALB and high APTT as important risk factors of postoperative RBC transfusion. ALB plasma concentrations is an indicator of nutritional status and hepatic function (28), and low preoperative ALB level reflects the malnutrition and anemia of patients before surgery (29), which might increase the risk of postoperative RBC transfusion. A prolonged preoperative APTT, which indicates the coagulation functions of patients (30), may be correlated to a high risk of intraoperative and postoperative bleeding, which leads to the requirement for RBC transfusion.

The type of operation and increased age were strongly associated with a higher risk of RBC transfusion after orthopedic surgery. Interestingly, a threshold effect was identified for the age of patients. Orthopedic patients aged over 60 years had a markedly increased risk of postoperative RBC transfusion compared with those under 60 years. Lenoir et al. (31) reported that age was the independent preoperative predictor of homologous RBC transfusion in patients undergoing elective spine surgery of France population. Patients aged over 50 years old had nearly 5-fold higher risk of transfusion compared with those with age under 50 years old. Moreover, Torres-Claramunt et al. (32) found that age over 60 years was a risk factor for postoperative transfusion in patients who underwent surgeries for degenerative conditions of the lumbar spine in the Spain population. These findings suggested that clinicians and surgeons might need to pay extra attention to the requirement of postoperative transfusion for elderly patients with orthopedic surgery. The threshold effect of RBC count was also identified. Orthopedic patients with RBC count under 4.0 × 10^12^/L had a growing risk of postoperative transfusion. And as expected, the relationship between the preoperative Hb level and the postoperative RBC transfusion was approximately linear. Patients with higher preoperative Hb level had a lower risk of postoperative RBC transfusion.

The correlation between BMI and the postoperative RBC transfusion showed a bimodal effect. Both underweight and overweight orthopedic patients had a higher risk of postoperative RBC transfusion. This finding is consistent with previous work. For example, Durand et al. (17) used a machine learning model to predict blood transfusion after adult spinal deformity surgery in the US population, and reported a bimodal risk profile. Patients with both low and high BMI were associated with an increased risk of transfusion. One potential explanation is that greater surgical exposure and complexity operation among patients with large BMI intensify the risk of intraoperative bleeding, thus predisposing the postoperative RBC transfusion, whereas patients with low BMI may have low blood volume, indicating that the blood loss threshold at RBC transfusion requirement would be lower than patients of greater size. However, Frisch et al. (33) reported that patients with increased BMI have lower rates of blood transfusion following total hip (THA) and knee (TKA) arthroplasty. Erben et al. (34) also concluded that patients with a higher BMI were potentially at lower risk for blood transfusion in the THA and TKA. These discrepancies could be attributed to the type of surgery or the different surgical plans among institutions. Further investigation with prospectively designed is warranted to clarify this issue.

The use of erythropoietin is usually proposed preoperatively to reduce blood transfusion in orthopedic surgery (35). However, in our study, the use of erythropoietin is correlated with the increased risk of postoperative RBC transfusion, indicating that medication intervention did not seem to achieve the desired effect or reflect a worse health state of patients. This result may be due to the fact that only a small proportion of patients with preoperative anemia (14.6%) were treated with medication, and erythropoietin was mostly prescribed to patients with severe anemia. Moreover, the treatment of erythropoietin was not early enough to take effect before surgery. Generally, erythropoietin should be initiated at least 1 month before surgery in patients at high risk of transfusion (36). However, the mean duration from admission to surgery was only 4.7 days in our cohort, much shorter than 1 month. Another retrospective cross-sectional study in Chinese adults reported that there was no significant association between preoperative erythropoietin and postoperative RBC transfusion (19). More studies are needed to clarify the relationship between erythropoietin and postoperative RBC transfusion.

Although the model derived from the machine learning algorithm presented good prediction performance, the inclusion of relatively too many numbers of predictors increased the complexity the of model and may limit their application. For the expedient application of the prediction model in clinical settings, we further developed a simpler risk score model with fewer variables (six variables) but similar prediction performance. All the continuous variables were categorized in the risk score system to conveniently perform prediction of postoperative RBC transfusion in orthopedic patients. The risk score model could be quickly deployed in most routine clinical situations or among outpatients without the need for a web-based platform or programming into the electronic health record system (37). In contrast, the machine learning prediction model may be more suitable for the requirement of accurate prediction.

This study has several strengths. First, our study included plentiful data consisting of multi-center datasets, which improved the generalization and reliability of the models. Second, different types of orthopedic operations were all included in the analysis. Thus, the machine learning prediction model had a wider range of applications, compared with those models only focusing on a specific type of surgery. Third, we used a novel interpretable machine learning approach to explain the output of the prediction model, which revealed the relationships between the identified risk factors and the outcome. Additionally, variables used in our risk score systems were only demographic characteristics, preoperative laboratory outcomes, and operation type, all of which could be easily obtained before surgery.

Several limitations of this study should be discussed. First, the exclusion of patients with missing critical data might induce some bias. However, the sample size of our study was large and the exclusion only accounted for a small fraction (<10%). Second, the surgeons at each hospital had different surgical plans, which might affect the results. However, the regular training of participating physicians and hospitals was implemented, which might reduce these differences. Third, although the test subset was divided for internal validation, the performance of our prediction model still needed further validation on the independent external dataset. Ultimately, our prediction model was focused on the orthopedic surgery patients of the Chinese population, so it might not applicable to other populations or patients of other surgeries.

## 5. Conclusion

On the basis of the interpretable machine learning framework, this large-scale multicenter retrospective cohort study identified novel modifiable risk factors and developed prediction models with good performance for RBC transfusion in patients undergoing orthopedic surgery. These findings may allow more precise identification of high-risk patients for optimal control of risk factors and achieve personalized RBC transfusion for orthopedic patients.

## Data Availability

The raw data supporting the conclusions of this article will be made available by the authors, without undue reservation.
