# Supplementary material for: Prediction of red blood cell transfusion after orthopedic surgery using an interpretable machine learning framework
Source: Front Surg. 2023 Mar 2;10:1047558. doi: 10.3389/fsurg.2023.1047558 (PMC10017874; doi:10.3389/fsurg.2023.1047558)
Supplement: Supplementary file 1 [file Datasheet1.docx]

Supplementary Material

# Supplementary Figures and Tables

## Supplementary Figures


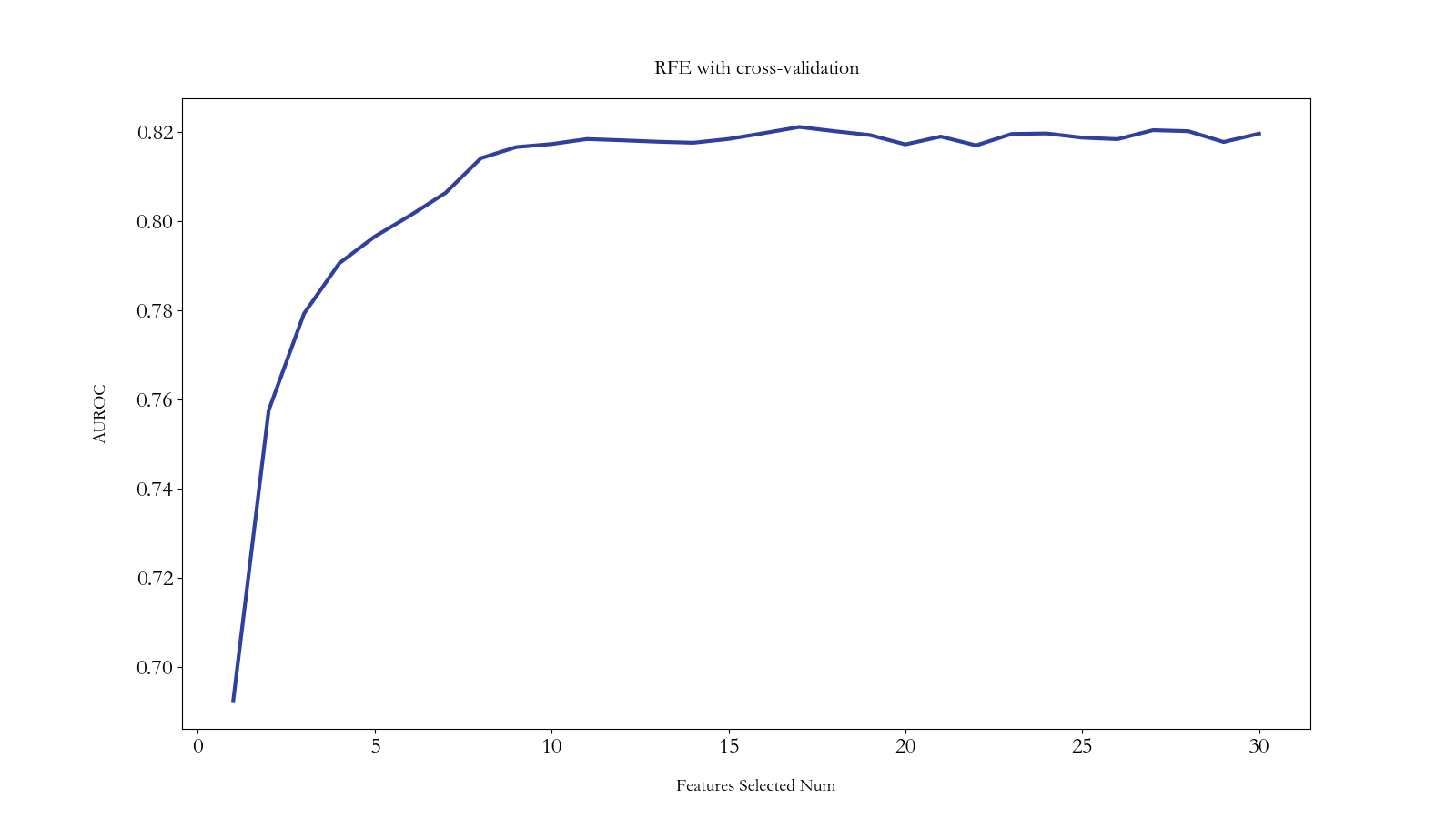


**Supplementary Figure 1.** Recursive Feature Elimination (RFE) Curve of the thirty features

The x-axis is the number of features in each subset and the y-axis indicates the corresponding AUC. The subset with the largest AUC is selected, which includes seventeen features. spaced. Please use a single paragraph for each legend and prepare the figures keeping in mind the PDF layout.


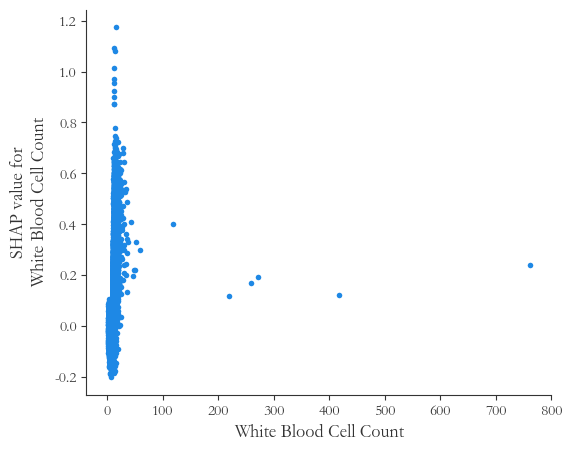

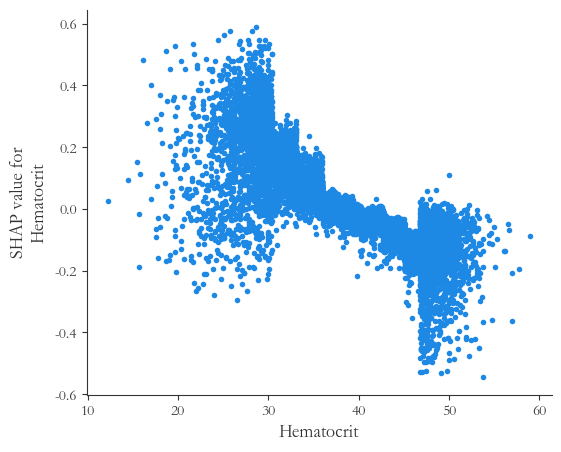


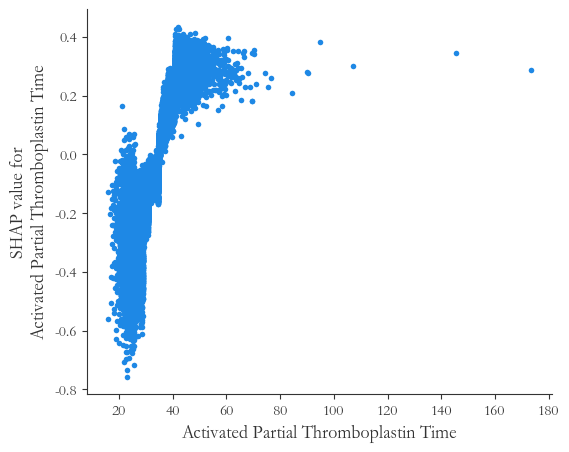

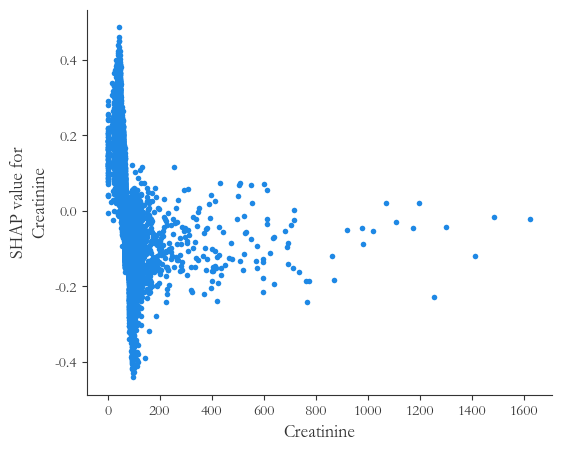


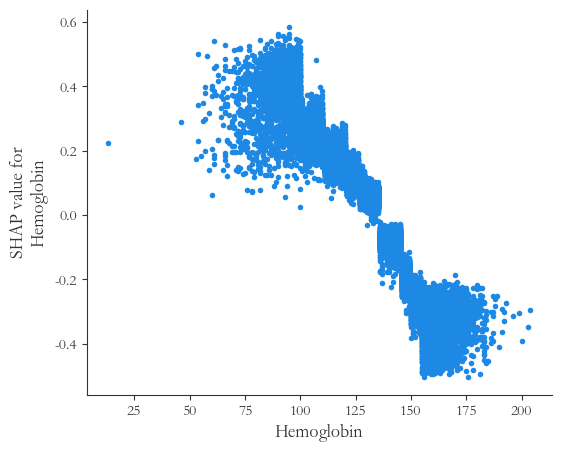

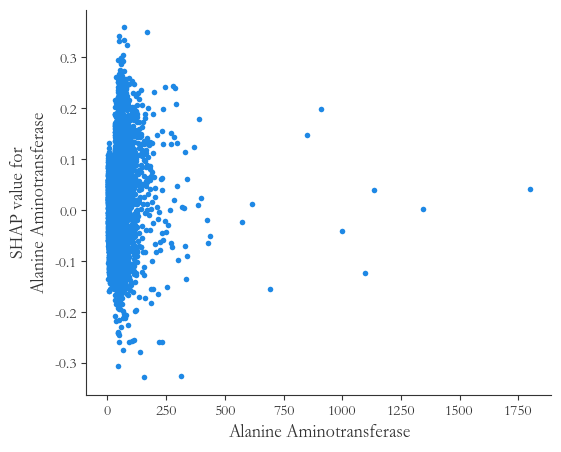


**Supplementary Figure 2.** SHAP partial dependence plots of the other predictors in the CatBoost model

Each plot shows how a single risk factor affects the outcome of the prediction model. SHAP values for specific factors exceed zero, representing an increased risk of postoperative RBC transfusion in orthopedic surgery patients. SHAP, SHapley Additive exPlanations.


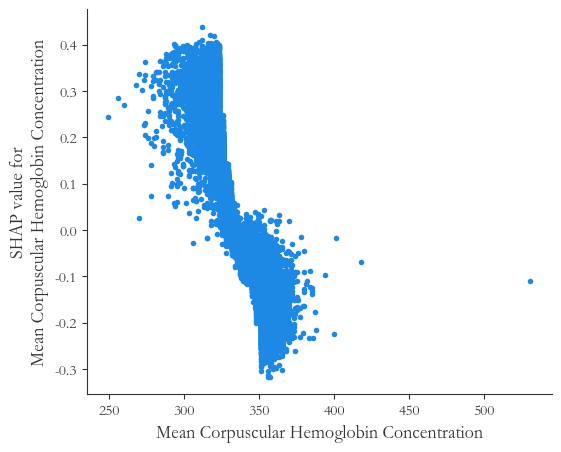

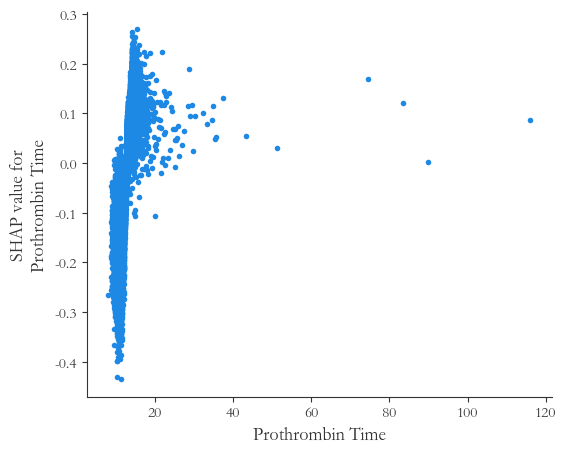

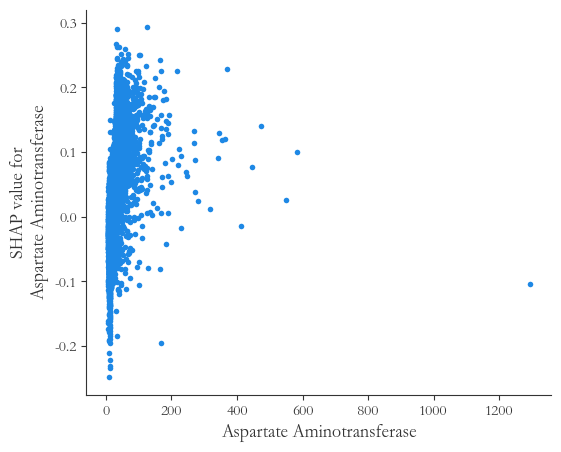

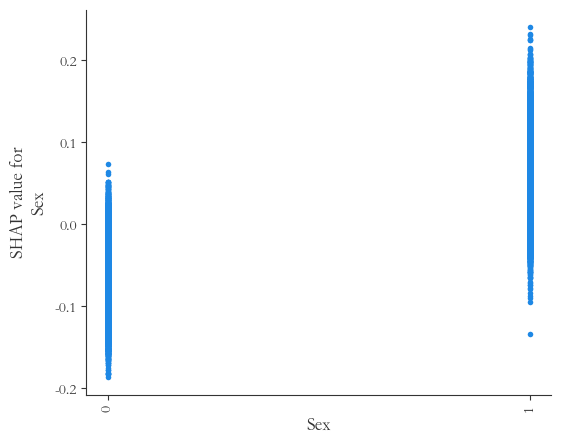

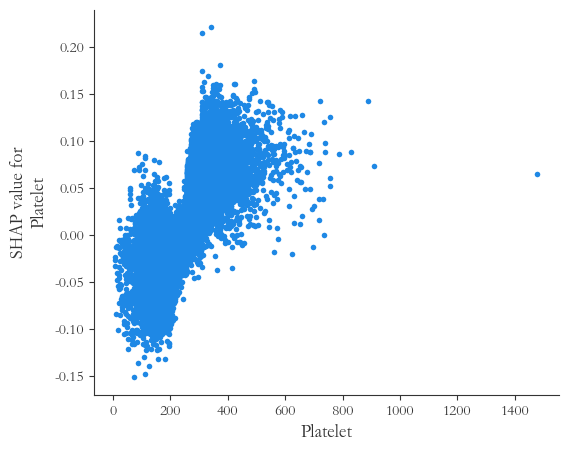


**Supplementary Figure 2.** (Continued)

Code of Sex: 0=Male; 1=Female.
